# Supplementary material for: A Thiopurine-like Mutagenic Process Defines TGCT Subtypes
Source: bioRxiv. 2025 Jun 12:2025.06.12.655573. Preprint. [Version 1] doi: 10.1101/2025.06.12.655573 (PMC12191103; doi:10.1101/2025.06.12.655573)

**Supplementary Fig. 1**

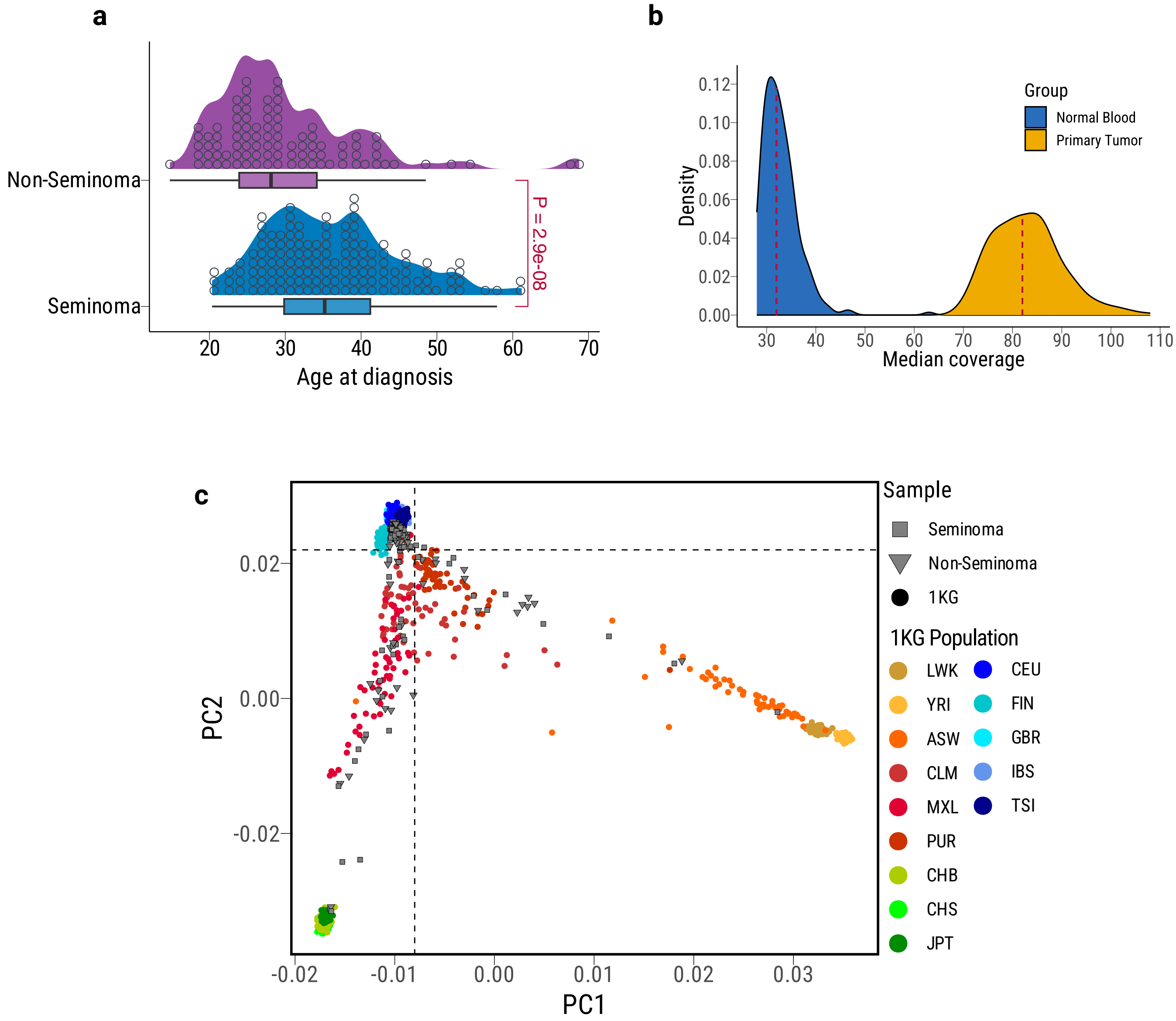

**Supplementary Fig. 2**

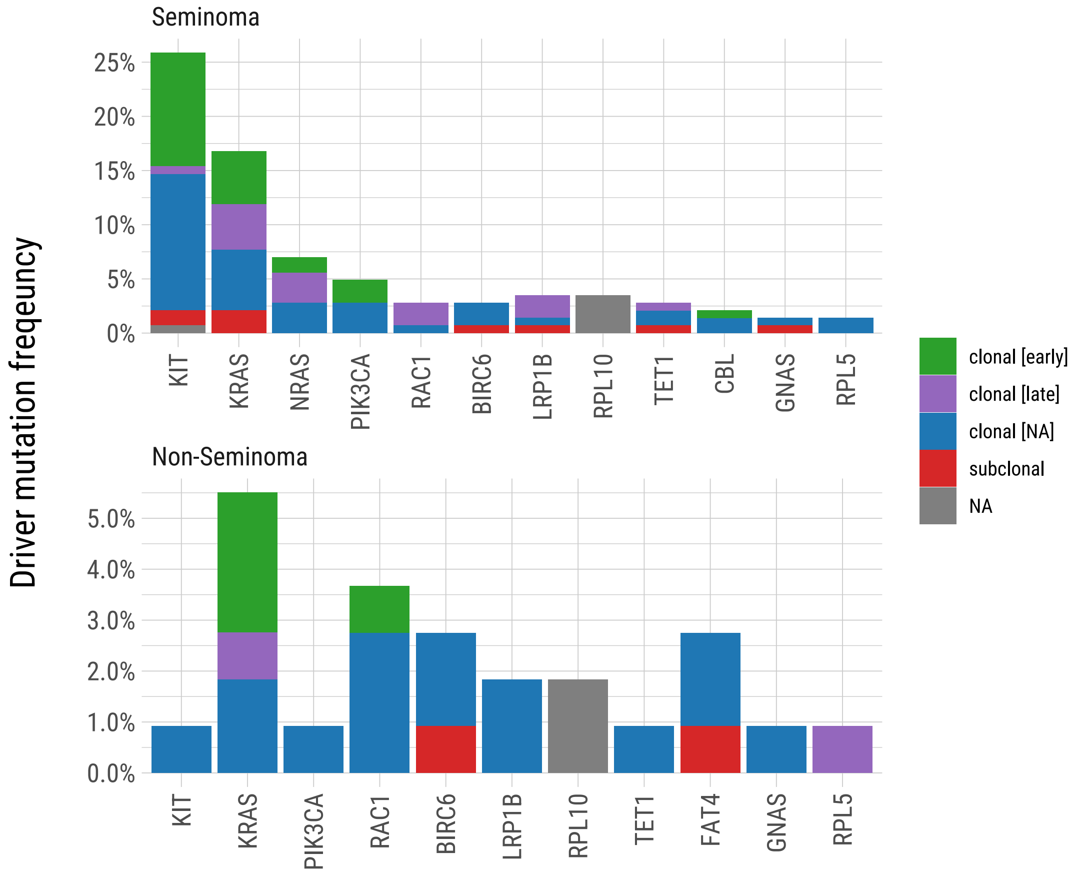

# Supplementary Fig. 3

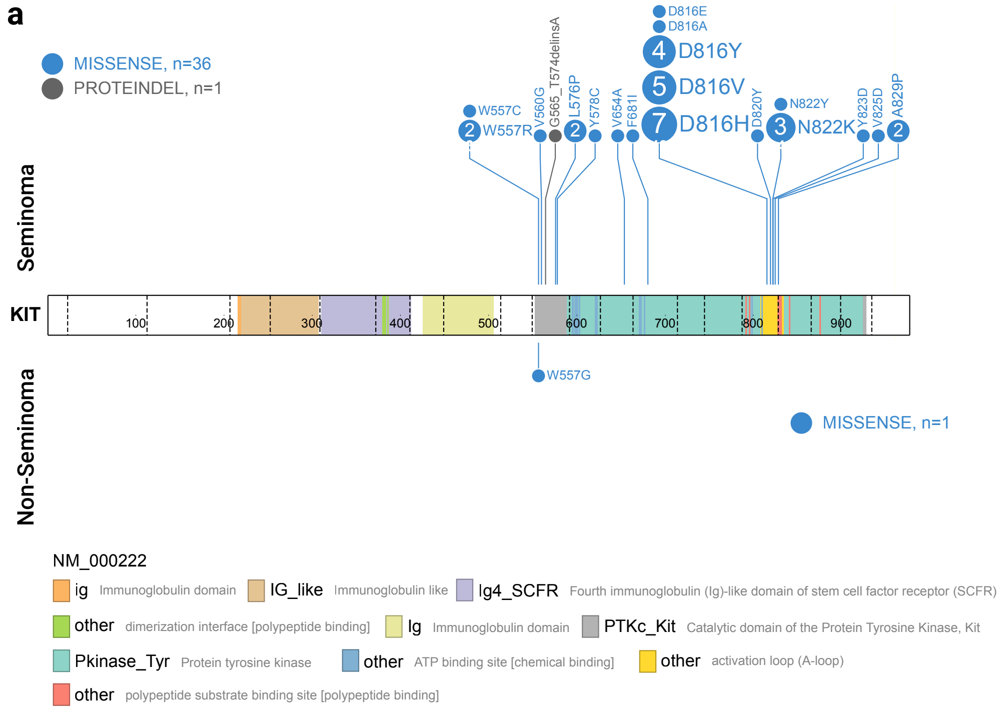


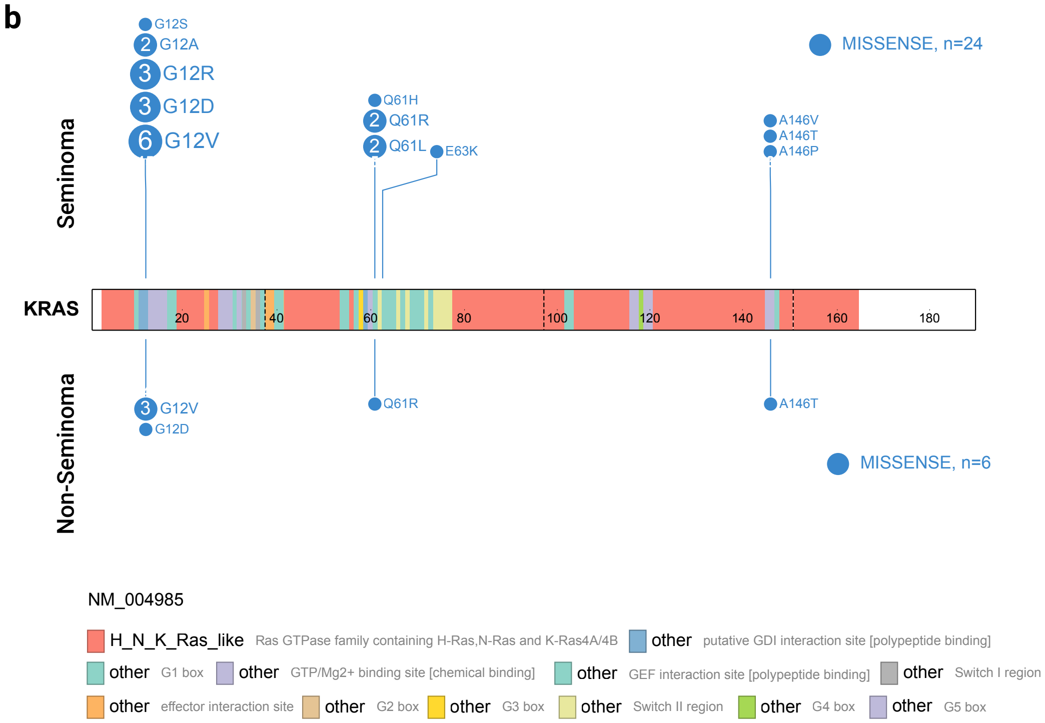

## Supplementary Fig. 4

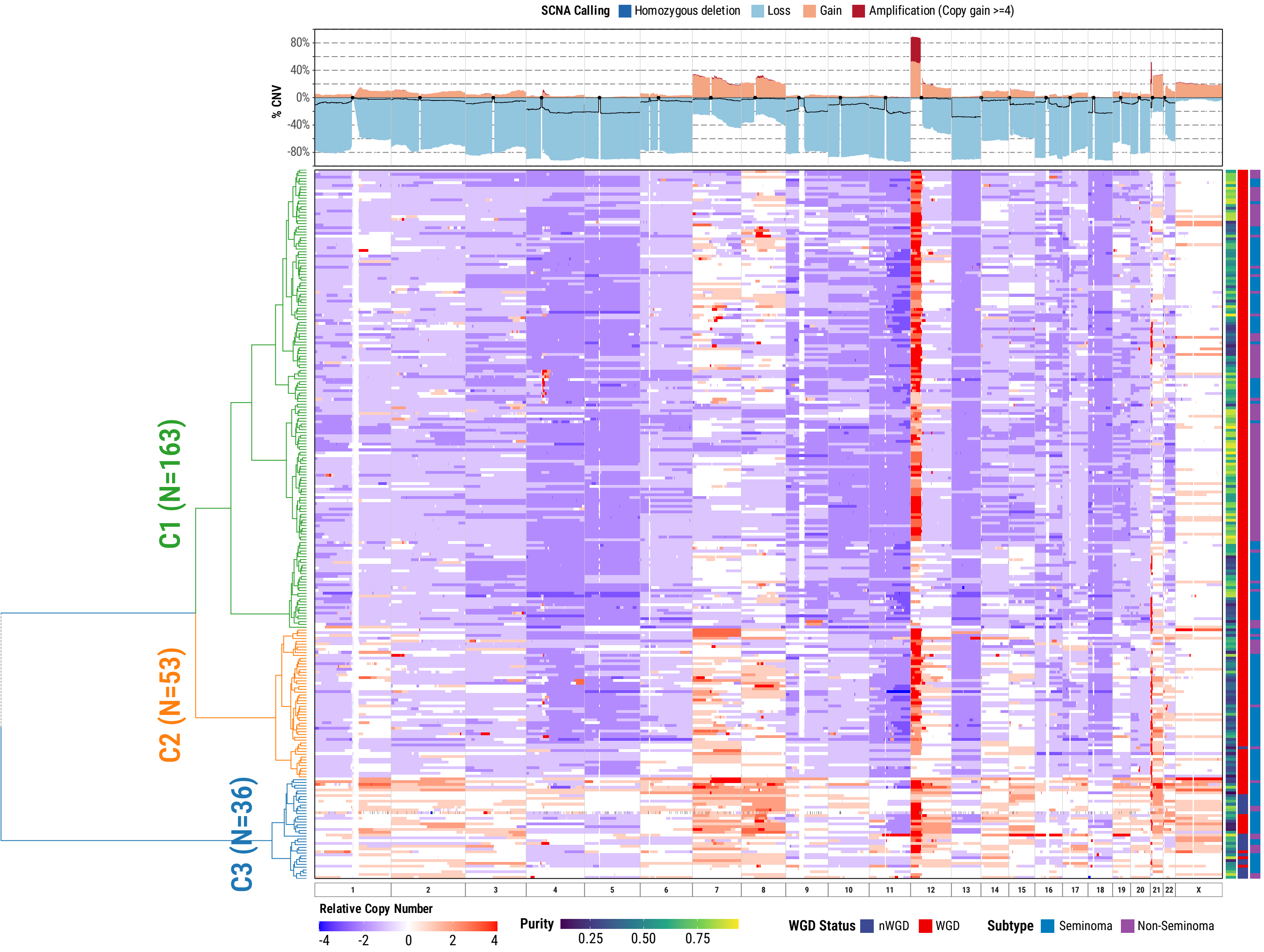

## Supplementary Fig. 5

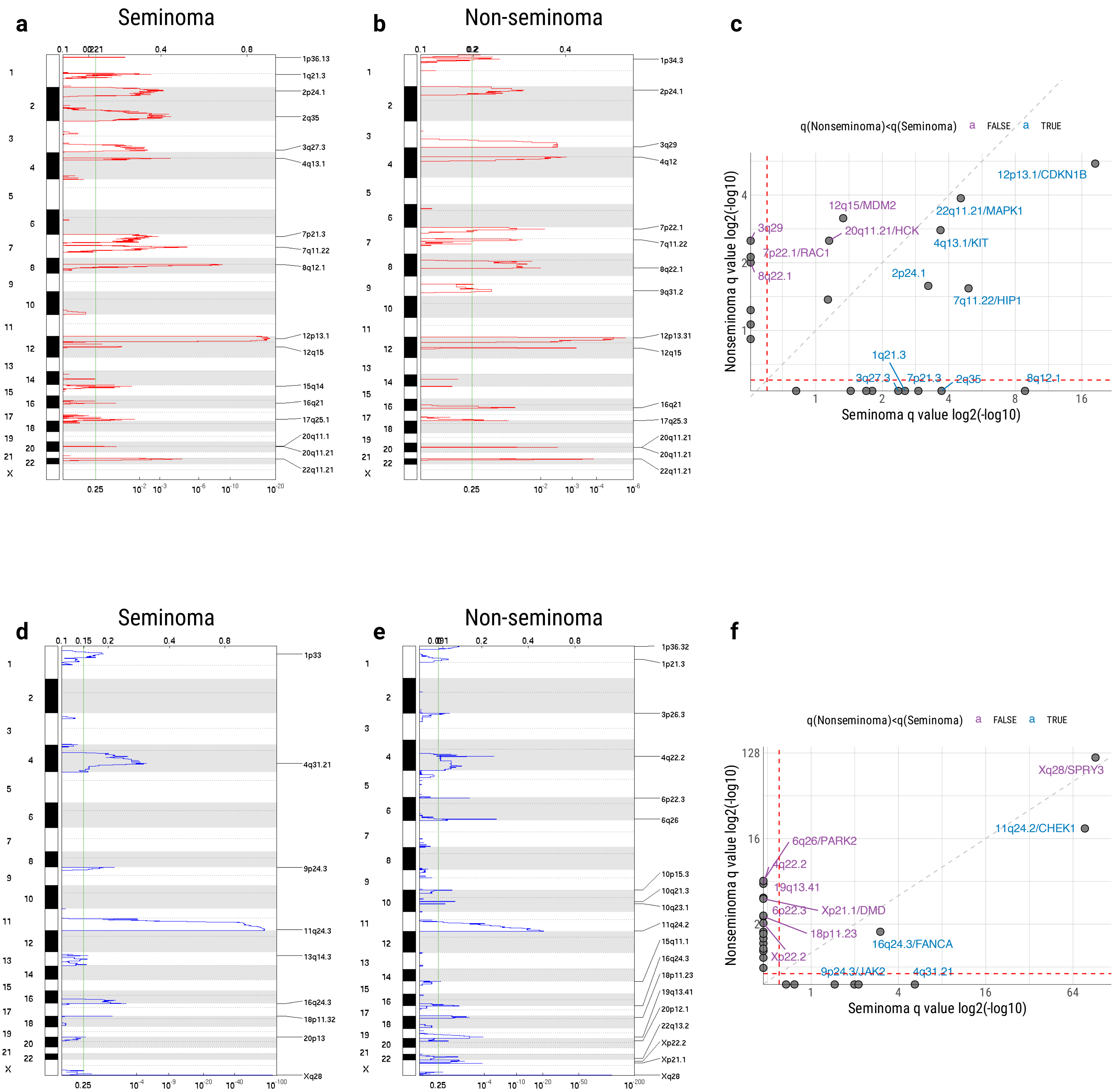

## Supplementary Fig. 6

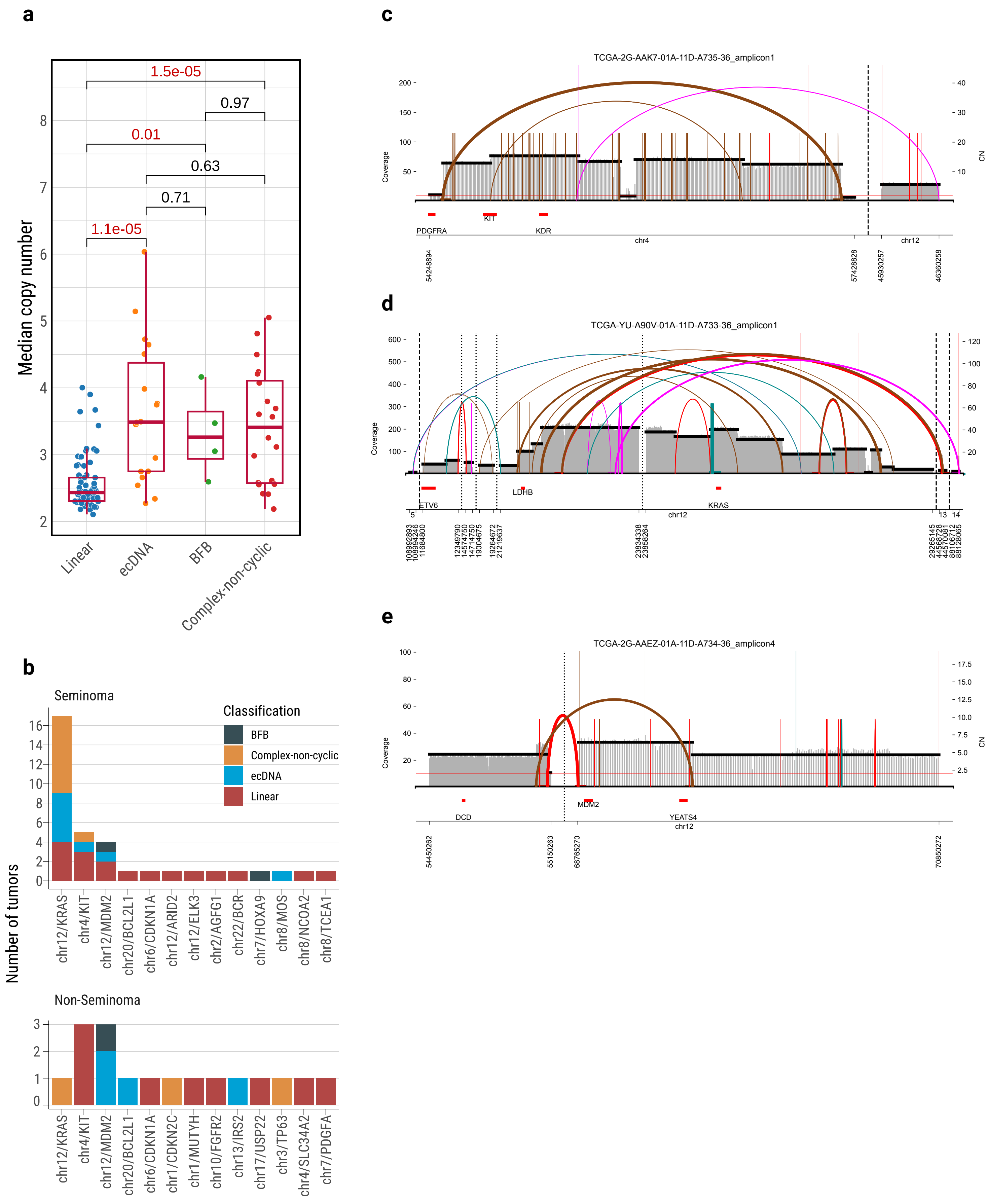

**Supplementary Fig. 7**

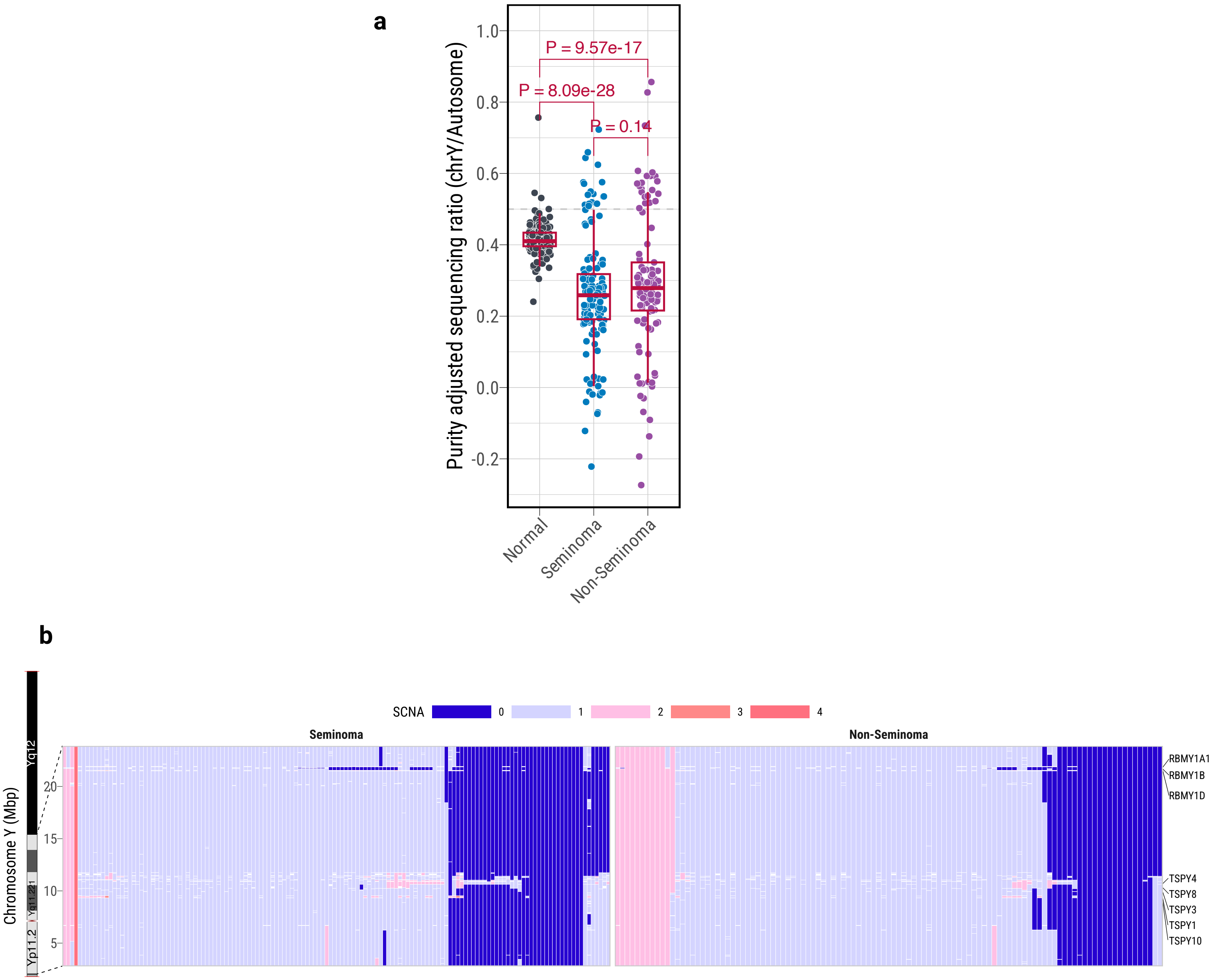

**Supplementary Fig. 8**

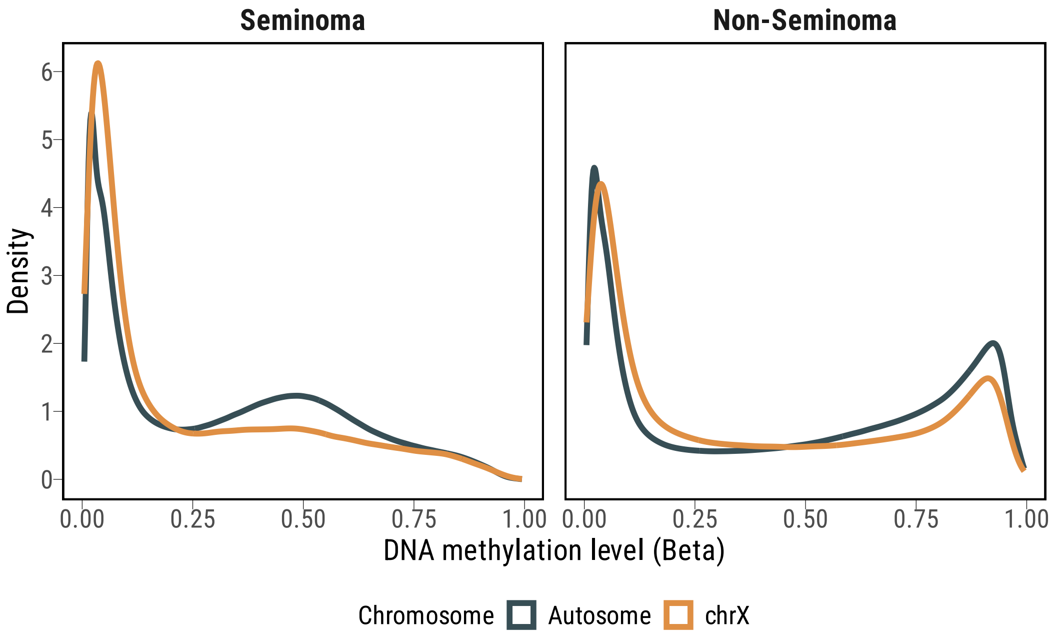

**Supplementary Fig. 9**

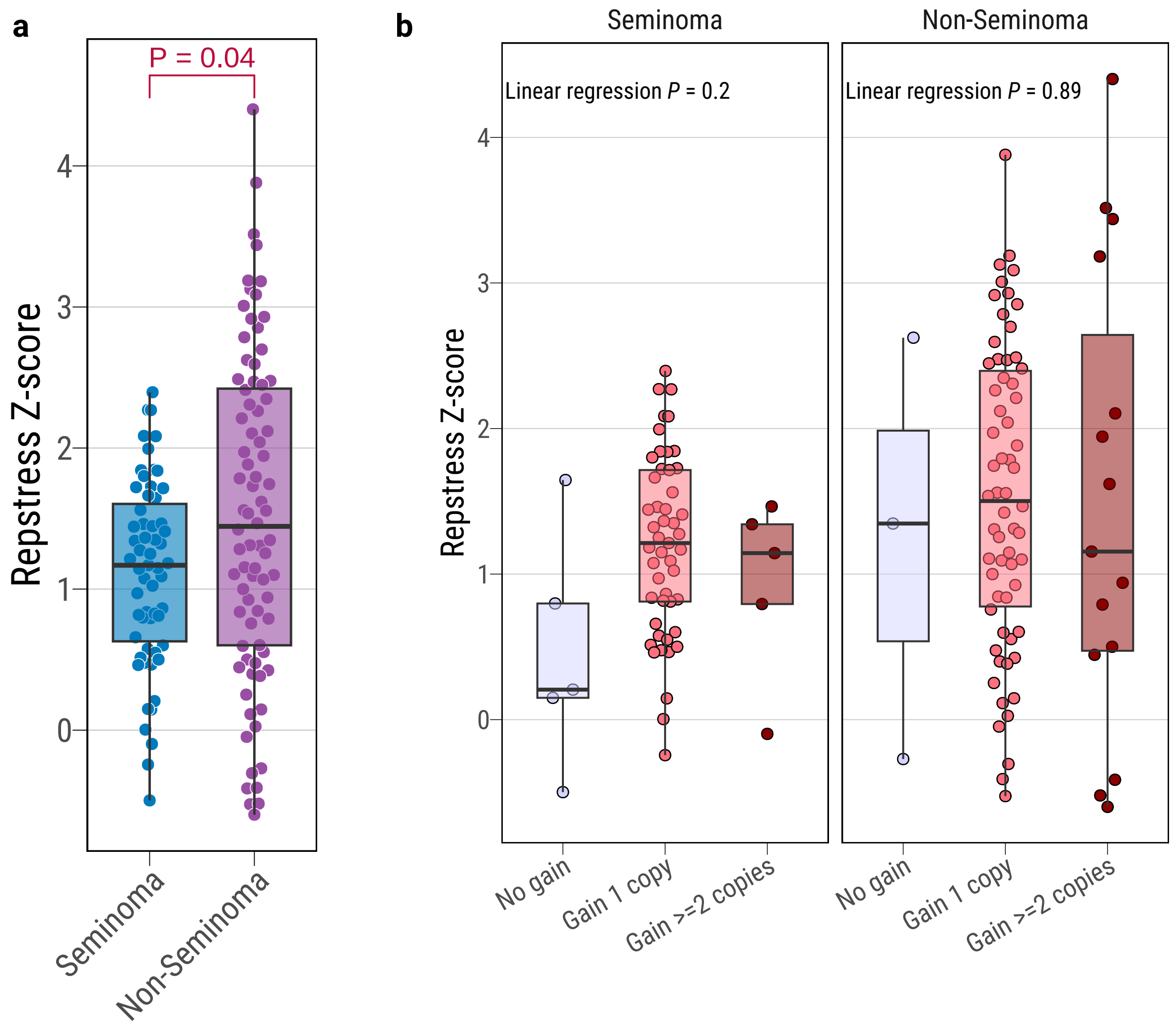

**Supplementary Fig. 10**

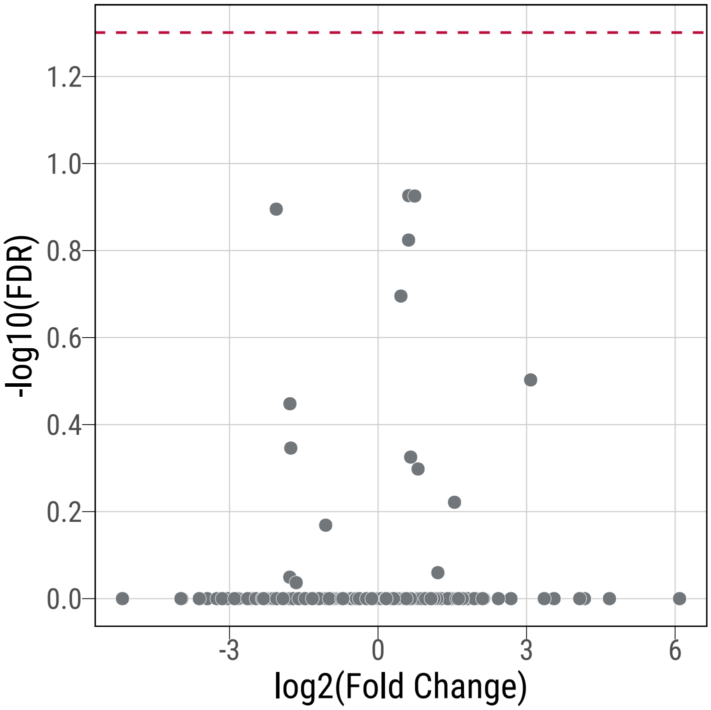

**Supplementary Fig. 11**

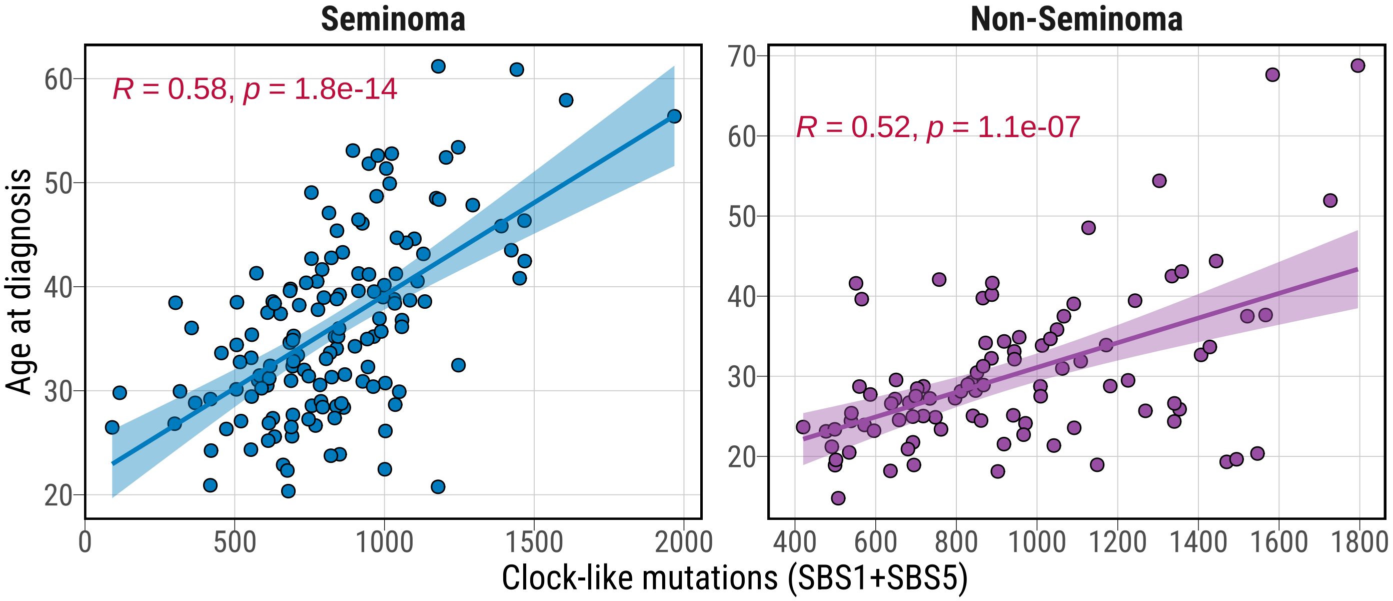

Supplementary Fig. 12

a

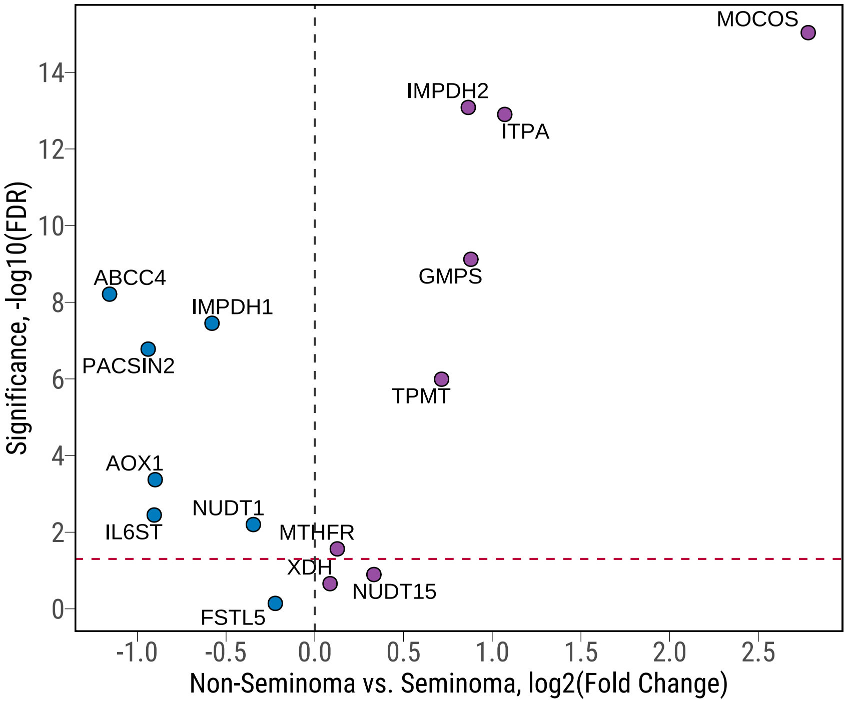


b

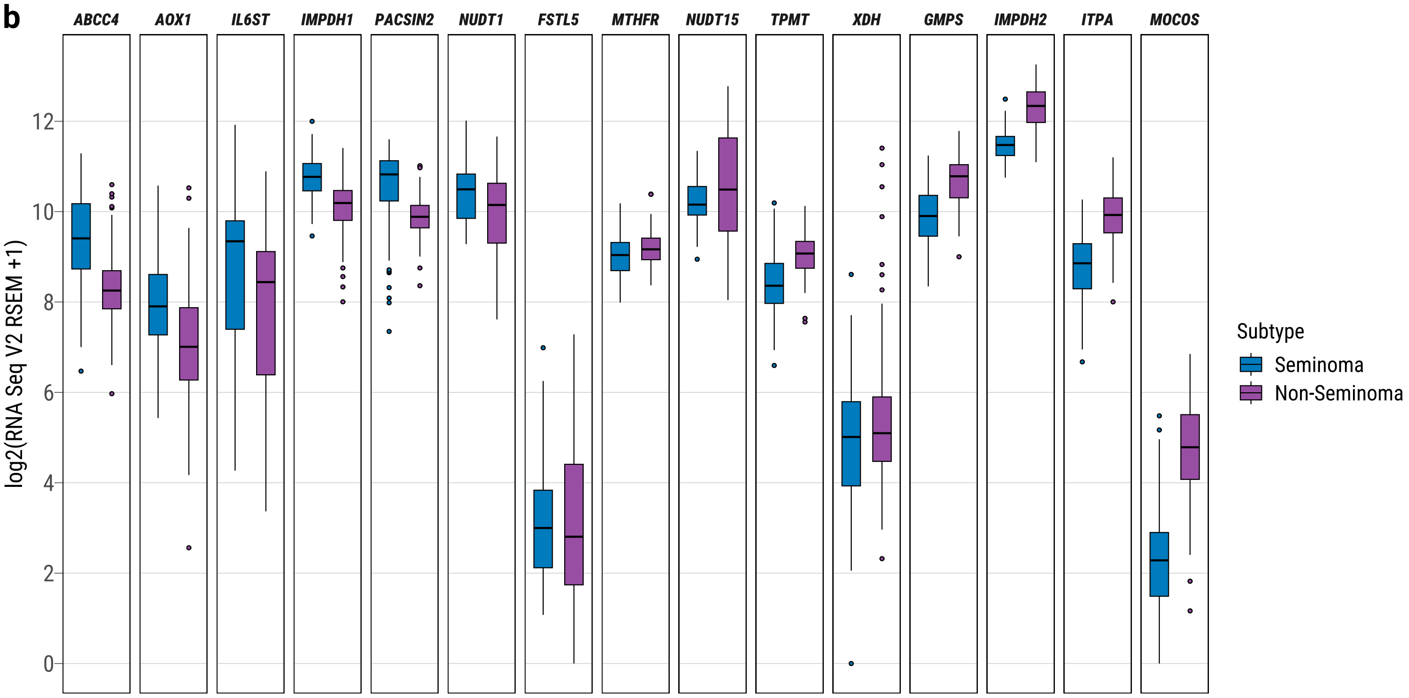

**Supplementary Fig. 13**

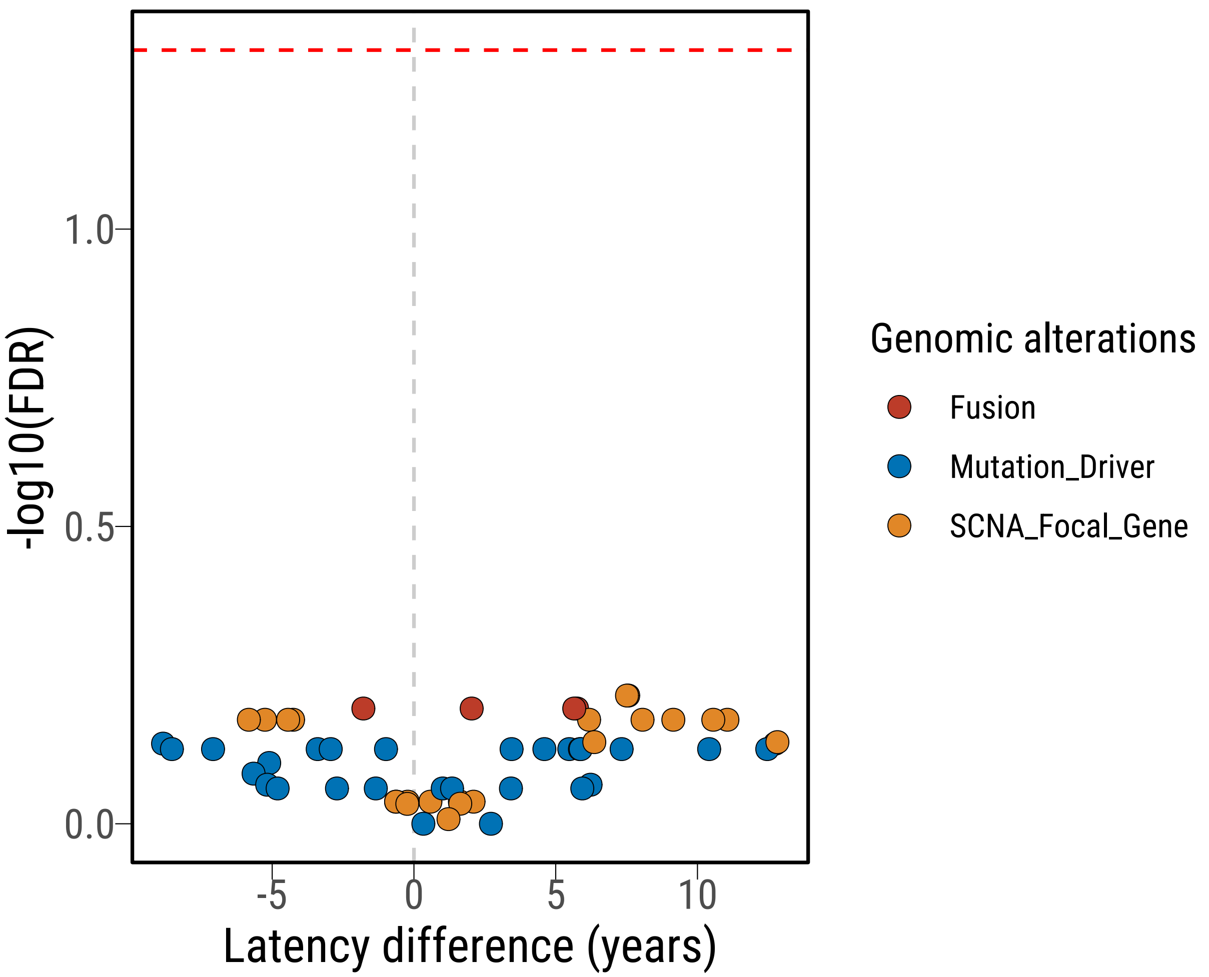

**Supplementary Fig. 14**

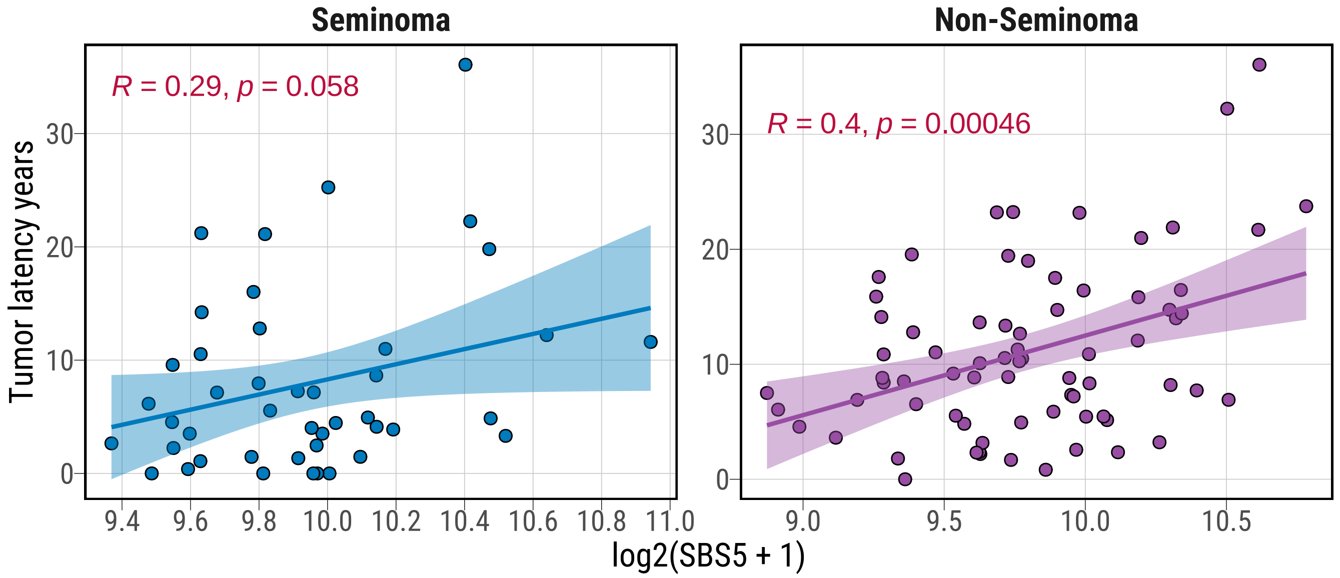

**Supplementary Fig. 15**

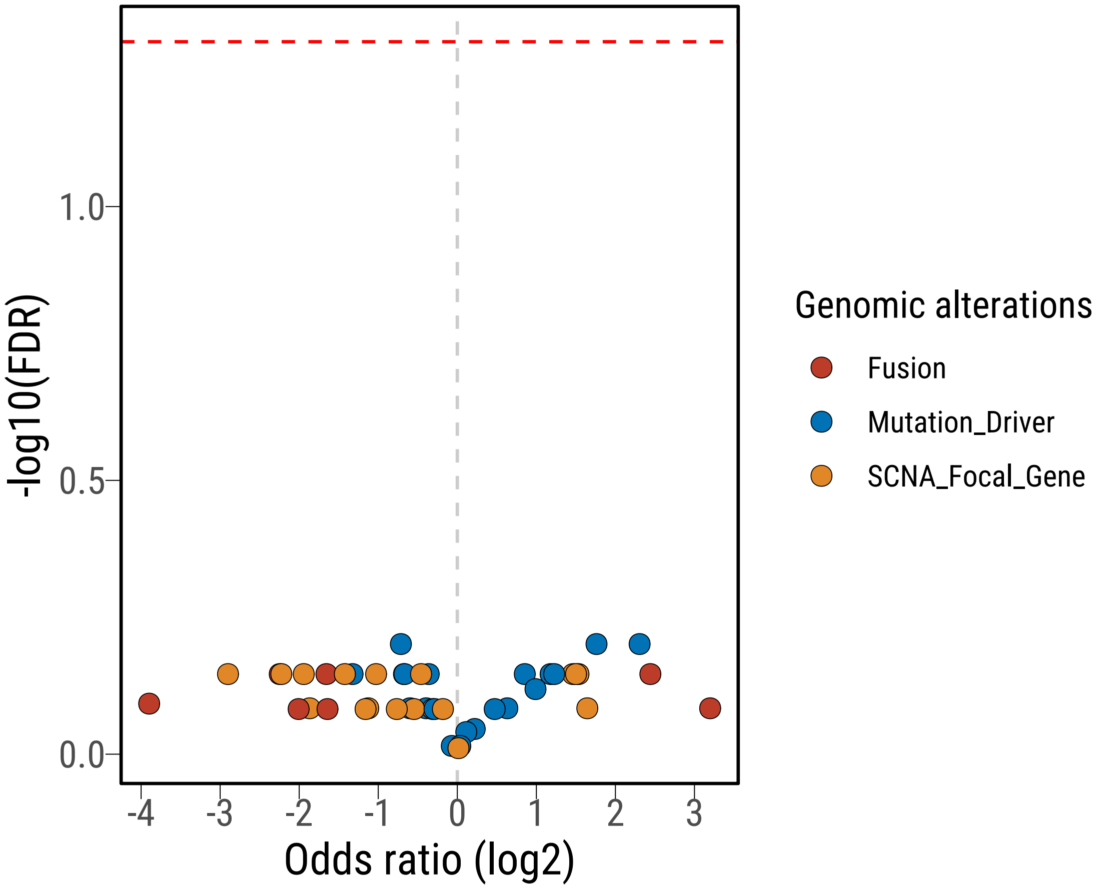

**Supplementary Fig. 16**

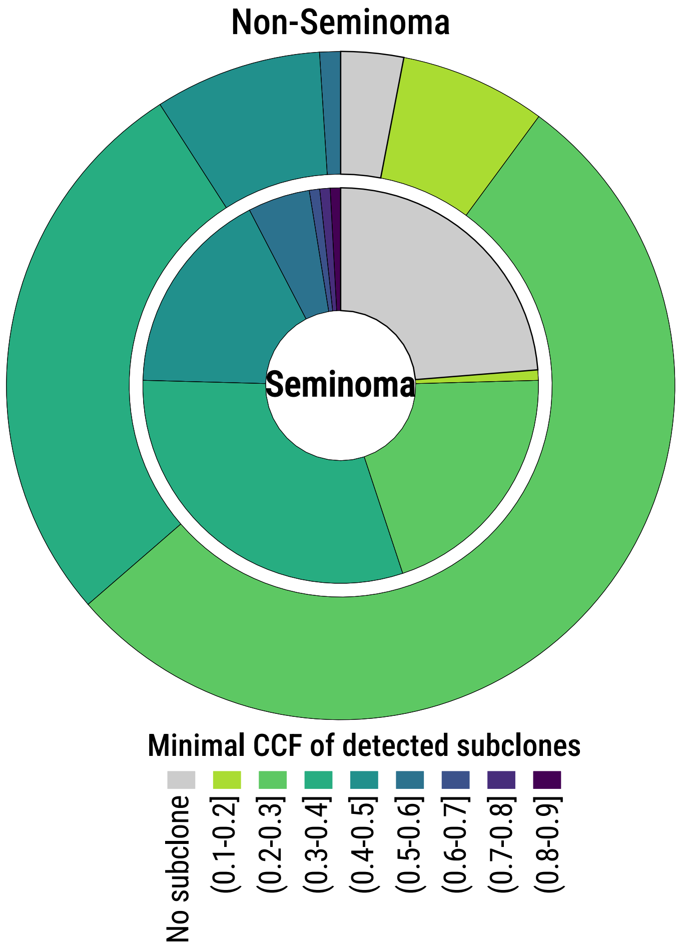

**Supplementary Fig. 17**

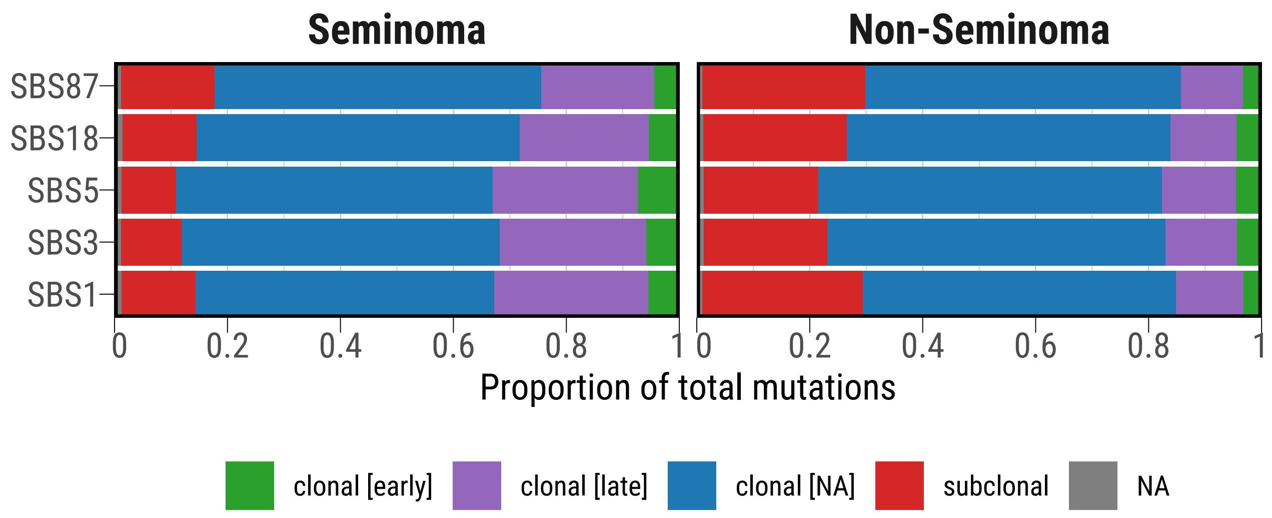

**Supplementary Fig. 18**

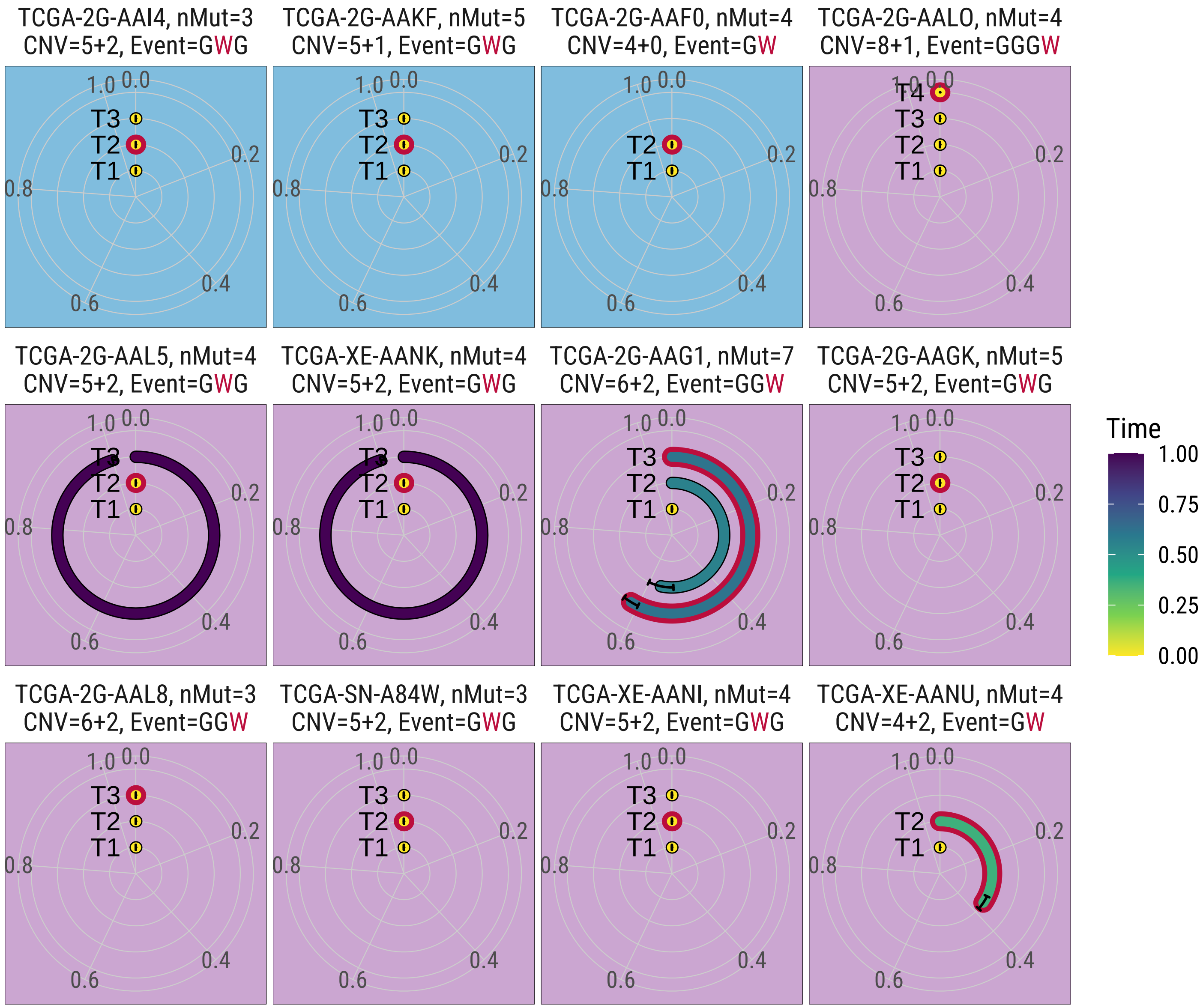

# Supplementary Fig. 19

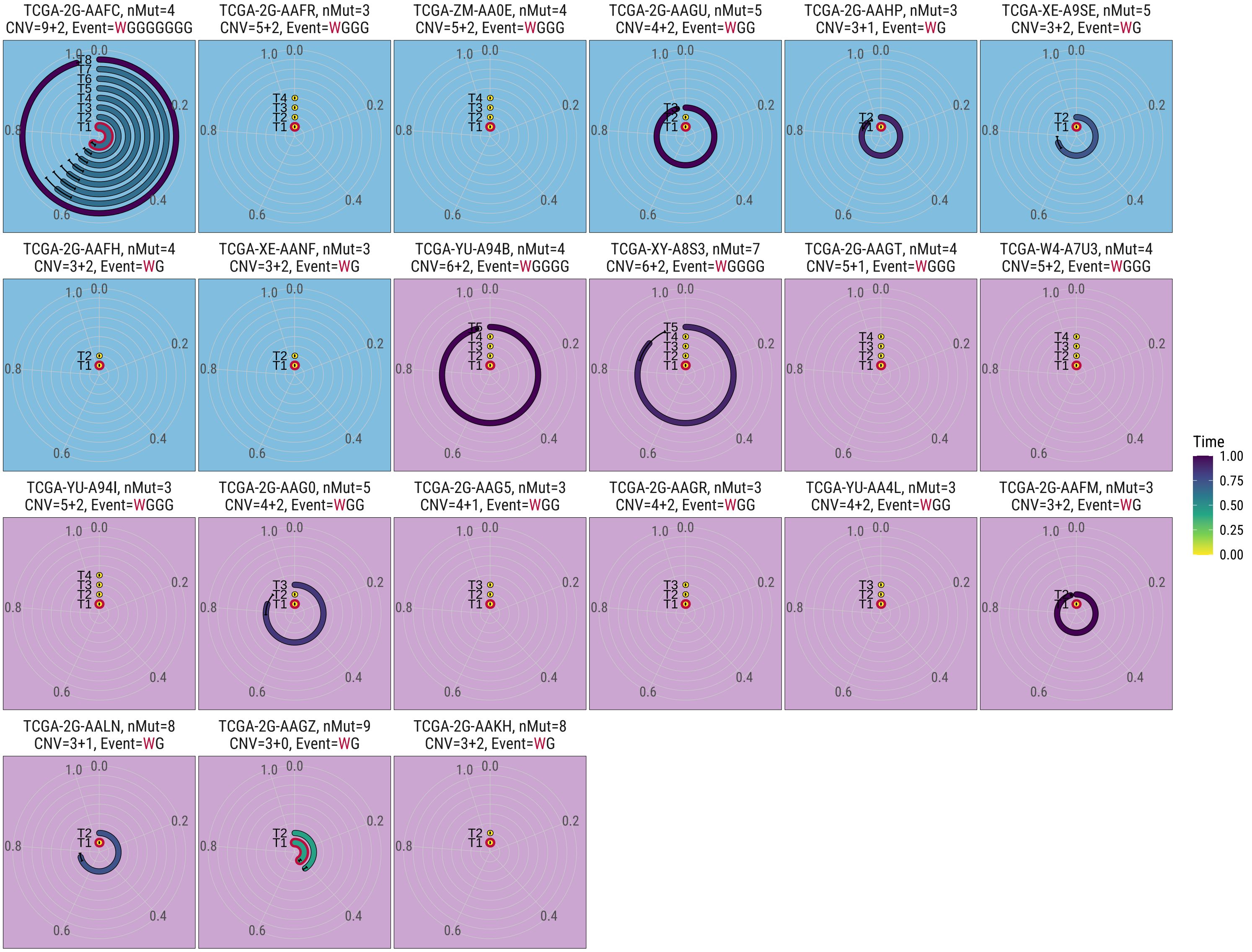

# Supplementary Fig. 20

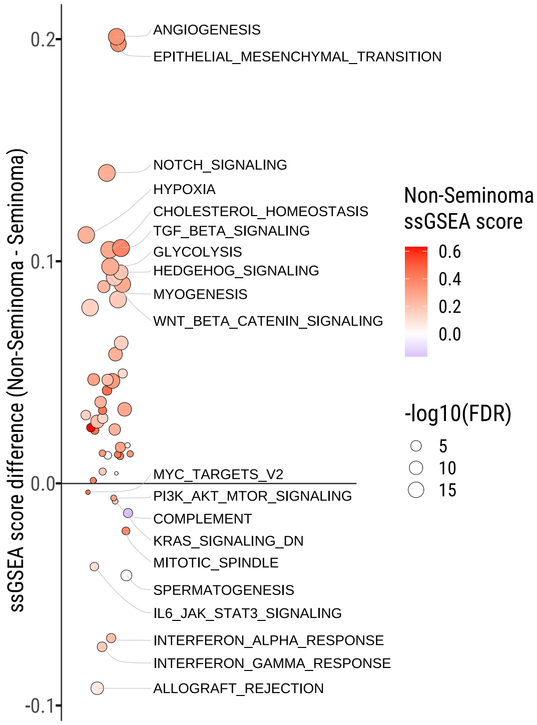


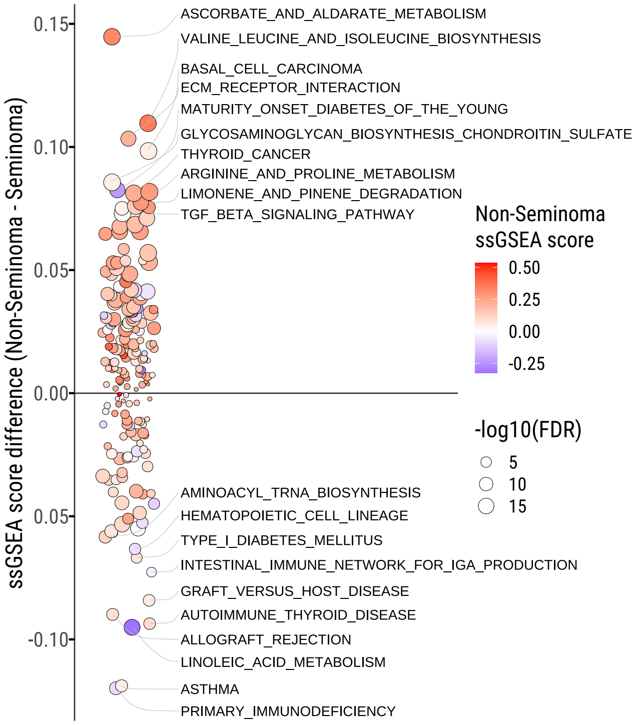

Supplement: Supplement 1 — Supplementary Fig. 1: Summary of clinical and sequencing characteristics for the TCGA TGCT cohort. (a) Comparison of age at diagnosis between non-seminomas and seminomas. P-value from two-sided Wilcoxon rank-sum test is shown. (b) Distribution of median WGS sequencing depth for tumor and matched normal samples. Median depth across samples of each type is indicated by a red vertical line. (c) Principal component analysis (PCA) for ancestry inference using 1000 Genomes reference samples. Supplementary Fig. 2: Stacked bar plot showing the timing of point mutations in recurrent driver genes in TGCTs. The majority of mutations occur early during tumor evolution in both seminomas and non-seminomas. Supplementary Fig. 3: Lollipop plots of non-synonymous mutations in the major TGCT driver genes KIT (a) and KRAS (b). Mutation counts are shown separately for seminomas (top) and non-seminomas (bottom), generated using ProteinPaint (https://proteinpaint.stjude.org/). Supplementary Fig. 4: Arm-level SCNA classification of TGCTs. Left, Unsupervised clustering of TGCT samples based on arm-level SCNA profiles and visualization of copy number is based on relative copy number: total copy number – ploidy (non-WGD = 2 and WGD = 4). The analysis revealed three major SCNA subgroups, designated C1, C2, and C3. Top, Frequency of SCNA events, including gains (red), losses (blue), and copy-neutral loss of heterozygosity (LOH; black line) for each chromosomal arm. Supplementary Fig. 5: GISTIC-based analysis of recurrent focal SCNAs for TCGT subtypes. (a-b) Significant focal amplifications in seminomas and non-seminomas, respectively. (c) Comparison of amplification significance (q-values) between subtypes. (d-e) Significant focal deletions in seminomas and non-seminomas, respectively. (f) Comparison of deletion significance between subtypes. Supplementary Fig. 6: Detection and classification of extrachromosomal DNA (ecDNA) in TGCTs. (a) Comparison of median copy number among different amplifi [file media-1.pdf]
